# Tongue conservation treatment for oral tongue squamous cell carcinoma with induction chemotherapy, surgery, and risk‐adapted adjuvant therapy: A phase II trial

**DOI:** 10.1002/cnr2.1456

**Published:** 2021-05-29

**Authors:** Tsung‐Lun Lee, Pei‐Yin Wei, Muh‐Hwa Yang, Peter Mu‐Hsin Chang, Ling‐Wei Wang, Shyh‐Kuan Tai

**Affiliations:** ^1^ Department of Otolaryngology National Yang Ming Chiao Tung University Taipei Taiwan; ^2^ Department of Otolaryngology Taipei Veterans General Hospital Taipei Taiwan; ^3^ Department of Otolaryngology Taoyuan General Hospital, Ministry of Health and Welfare Taoyuan Taiwan; ^4^ Infection and Immunity Research Center National Yang Ming Chiao Tung University Taipei Taiwan; ^5^ Oncology, Division of Medical Oncology Taipei Veterans General Hospital Taipei Taiwan; ^6^ Institute of Clinical Medicine National Yang Ming Chiao Tung University Taipei Taiwan; ^7^ Oncology, Division of Radiation Oncology Taipei Veterans General Hospital Taipei Taiwan

**Keywords:** complete pathologic response, induction chemotherapy, Oral tongue squamous cell carcinoma, risk‐adapted adjuvant therapy, tongue conservation surgery

## Abstract

**Background:**

To assess the feasibility of tongue conservation treatment with induction chemotherapy (ICT), tongue conservation surgery, and risk‐adapted postoperative adjuvant therapy in oral tongue squamous cell carcinoma (OTSCC).

**Methods:**

Patients with newly diagnosed OTSCC cT2‐4 N0‐2 M0 were recruited. The ICT with a regimen of docetaxel, cisplatin, and oral tegafur/uracil (DCU) was administrated every 21 days. After the first cycle of ICT (DCU1), patients with a more than 30% decrease in the longest diameter of primary tumor underwent a second cycle of ICT (DCU2). Tongue conservation surgery was performed after ICT, and risk‐adapted adjuvant therapy was organized based on pathological features.

**Results:**

From July 2011 to December 2015, a total of 23 patients were enrolled, 87% of whom were classified as stage III–IV. Clinical responders to DCU1 and DCU2 were determined in 90.5% (19/21) and 88.2% (15/17) of patients. Tongue conservation surgery was performed in 16 responders to ICT. Only one patient had a positive margin (6.3%), and a complete pathologic response was achieved in eight patients (50%). Only one patient developed local recurrence after a median follow‐up of 58.6 months (range, 7.9–105.2). The 5‐year overall survival (0% vs. 87.5%, *P* = 0.001) and disease‐specific survival (0% vs. 93.3%, *P* = 0.000) were significantly different between the DCU1 nonresponders and responders.

**Conclusion:**

Tongue conservation treatment with ICT, followed by conservation surgery and risk‐adapted adjuvant therapy, is feasible for patients with OTSCC who are good responders to ICT. However, the outcomes of nonresponders are dismal. Further study in a larger patient population is warranted.

## 1 INTRODUCTION

Oral squamous cell carcinoma (OSCC) is the sixth most common malignancy in Taiwan, with over 5000 new cases annually. Oral tongue squamous cell carcinoma (OTSCC) is a major oral cavity cancer,^1^ and radical surgery is the standard treatment for patients with OTSCC.^2^ Early T1–2 tumors can be treated by transoral excision, while larger T3–4 tumors may require mandibulotomy, mandibulectomy, or flap reconstruction. In addition, postoperative adjuvant radiotherapy or chemoradiotherapy (POCCRT) is indicated for patients with advanced T3–4 tumors, cervical lymph node metastasis, or adverse pathologic features. Such intensive treatment for OTSCC inevitably leads to functional sequelae.

Temporo‐spatially coordinated motility of the oral tongue is critical for normal swallow and speech functions. Greater volumetric or motility loss of oral tongue tissue after surgery can lead to poor quality of life with profound swallowing and speech impairments, especially in patients with large tumors and adjuvant radiation.^3^ Despite the availability of modern free tissue transfer reconstruction, the benefit for residual tongue functions is still controversial.^2^, ^4^ Conserving the oral tongue without jeopardizing tumor control is the primary treatment goal for OTSCC, but is not easily attained. Although organ preservation with chemoradiotherapy has been widely applied for head and neck cancers in the past two decades, patients with resectable OTSCC are usually excluded due to concerns about poorer responses and outcomes compared to radical surgery.^5^, ^6^

Induction chemotherapy (ICT) has been applied as an important component in the treatment of various advanced human cancers, including cancers of the rectum, cervix, and breast. For organ or tissue conservation purposes, and under appropriate circumstances, limited surgical excision of residual tumors after ICT is performed, followed by adjuvant therapy. In cervical cancers, the possibility of attempting a less extensive surgery after ICT has been investigated to improve the quality of life.^7^ Breast conservation treatment with ICT has become one of the standard treatments for breast cancer.^8^, ^9^ The main purpose of ICT is to downsize large primary breast tumors (> 3 cm) for breast‐conserving surgery, which involves resection of the gross residual tumor mass with a safe margin, instead of the original tumor extent.^10^, ^11^ A similar concept of organ or tissue conservation treatment with ICT has not yet been investigated in OSCC. For locally advanced head and neck cancers, a combination of docetaxel, cisplatin, and fluorouracil (TPF) has been shown by TAX323 and TAX324 studies to be the most effective ICT regimen. ICT consisting of paclitaxel and carboplatin with cetuximab is another feasible, effective, and well‐tolerated regimen.^12^ However, all aforementioned studies only included a small number of OSCC patients,^12^, ^13^, ^14^, ^15^ and the benefits of ICT in OSCC remain unclear.^16^, ^17^, ^18^, ^19^, ^20^ Because TPF has been shown to be superior to PF in randomized trials, for reasons of tolerance in Asian patients, we modified it to the docetaxel, cisplatin and oral tegafur/uracil (DCU) regimen, as published earlier in Asian papers.^21^, ^22^ We conducted this phase II study to assess the feasibility and safety of ICT, followed by tongue conservation surgery and risk‐adapted postoperative adjuvant therapy in the management of OTSCC.

## 2 MATERIALS AND METHODS

### 2.1 Study design

This was an open‐label, noncomparative phase II trial to evaluate the feasibility of tongue conservation treatment comprising ICT, tongue conservation surgery, and risk‐adapted adjuvant chemoradiotherapy. The primary end point was the response to ICT, and the secondary endpoints included the oncologic controls and survival. The sample size was calculated based on the two‐stage design by Simon.^23^ The first step was planned to include 11 patients, and if >7 responders were recorded, an additional 39 patients would be enrolled. However, the study was prematurely closed due to slow patient recruitment. The study protocol is registered in ClinicalTrials.gov (No. NCT03161548), and was approved by the Institutional Review Board of Taipei Veterans General Hospital.

### 2.2 Patients

Between July 2011 and December 2015, patients with newly diagnosed and histologically proven OTSCC, who were to receive curative treatment at Taipei Veterans General Hospital, a tertiary referral medical center, were screened. The inclusion criteria were as follows^1^: cT2‐4, N0‐2M0 by clinical and radiographic examinations^2^; postoperative tongue defect >4 cm by initial surgical planning^3^; age between 20 and 70 years^4^; ECOG performance status of 0–1^5^; adequate hematopoietic, hepatic, and renal functions; and^6^ signed informed consent. Patients were excluded if they had any of the following conditions^1^: history of head and neck or esophageal cancer^2^; prior head and neck chemoradiotherapy^3^; synchronous cancer history within 6 months^4^; tumor invasion to the mandible, tonsils, or > 1/3 base of tongue; and^5^ N3 or M1 disease. Patients who had a history of previous tongue surgery, who had developed distant metastasis or those who were physically or mentally unfit were also excluded.

Comprehensive pretreatment evaluation was performed and included physical examination with photographic documentation, endoscopy, computed tomography (CT) or magnetic resonance imaging, chest X‐ray or chest CT, and routine laboratory studies. Patients were staged in the 3 weeks prior to recruitment using the seventh edition of the AJCC TNM staging system.^24^ The original surgical planning upon screening, including the approach and reconstruction, was recorded by the responsible surgeons.

### 2.3 Induction chemotherapy, tumor response, and safety profile of induction chemotherapy

**FIGURE 1:**
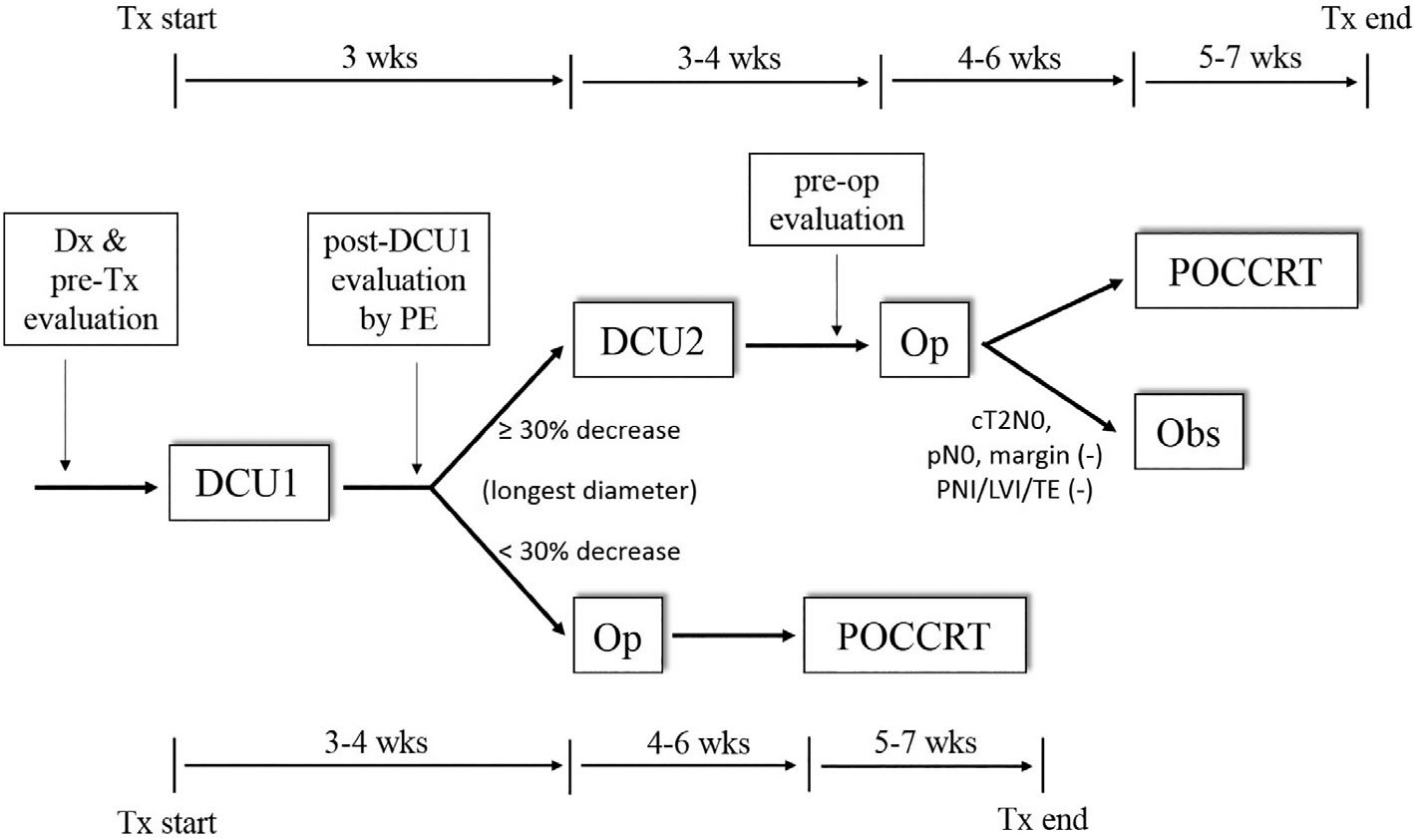
Flowchart of the Phase II trial for oral tongue squamous cell carcinoma. Induction chemotherapy, followed by conservative surgery and risk‐adapted adjuvant therapy

The treatment algorithm and schema are shown in Figure 1. The DCU regimen was used for ICT, which consisted of intravenous infusion of docetaxel 36 mg/m^2^ over 1 h, followed by intravenous infusion of cisplatin 30 mg/m^2^ over 1 h on day 1 and day 8, and oral tegafur/uracil (UFUR) 300 mg/m^2^/d plus leucovorin 90 mg/day on days 1–14; each cycle of ICT lasted 21 days. After the first cycle of ICT (DCU1), the tumor response was assessed by meticulous palpation on day 20. When a reduction in tumor volume of more than 30% was achieved in the longest diameter of the oral tongue tumor compared to pretreatment documentation, patients underwent the second cycle of ICT (DCU2). Nonresponders to DCU1 (< 30% decrease in the longest diameter) were arranged to undergo immediate surgery and POCCRT. The adverse events (AE) of chemotherapy were graded by the NCI Common Terminology Criteria for Adverse Events (CTCAE), version 4.0, and tumor responses after DCU2 were evaluated by RECIST criteria.^25^

### 2.4 Surgery for residual tongue tumor

Surgery, including excision of the tongue tumor and neck dissection, was performed 3–4 weeks after the start of the last cycle of ICT. The surgical approach and the need for flap reconstruction were determined by the clinical judgment of the surgeons responsible for tissue conservation. The principle of excision was based on the residual tumor or induration after ICT with a safe margin of at least 1 cm. In pathological examination, all residual tumors were paraffin‐embedded, and routine serial sections were taken at 5 mm intervals.

### 2.5 Risk‐adapted postoperative adjuvant concurrent chemoradiotherapy

POCCRT was arranged based on a risk‐adapted consideration. POCCRT was not indicated if fulfilling all following criteria^1^: cT2N0 at initial presentation^2^; ypN0^3^; negative margin without perineural invasion or lymphovascular invasion. POCCRT was started 4–6 weeks after surgery with the intensity modulation radiotherapy technique, with a 2 Gy once‐daily fraction size, 5 days a week. The medium‐risk clinical target volume (CTV) of 60 Gy covered the tumor bed and regions of grossly involved neck lymph nodes. The low‐risk CTV of 54 Gy covered other regions felt to be at risk of microscopic diseases, such as the contralateral neck. The high‐risk CTV of 66 Gy covered regions at high‐risk of recurrence, such as close or positive margins. Chemotherapy was given concomitantly, with weekly cisplatin 25 mg/m^2^ administered intravenously for 4 h, for a total of 6 cycles, and oral UFUR 200 mg given twice a day throughout the whole course of POCCRT.

### 2.6 Statistical analysis

All analyzes were performed using the Statistical Package of Social Sciences software version 17.0 (SPSS, Inc., Chicago, IL). Descriptive statistics were used for most primary and secondary end points. Overall survival (OS) was defined as the interval between the start of ICT and the date of death or last contact. Disease‐specific survival (DSS) was defined as the interval between the start of ICT and the date of death from the index tumor or treatment‐related events. Kaplan‐Meier analysis and the log‐rank test were used for survival analyzes. All tests were two‐sided, and results were considered significant at *P* < 0.05.

## 3 RESULTS

### 3.1 Patient characteristics and treatment course

**FIGURE 2:**
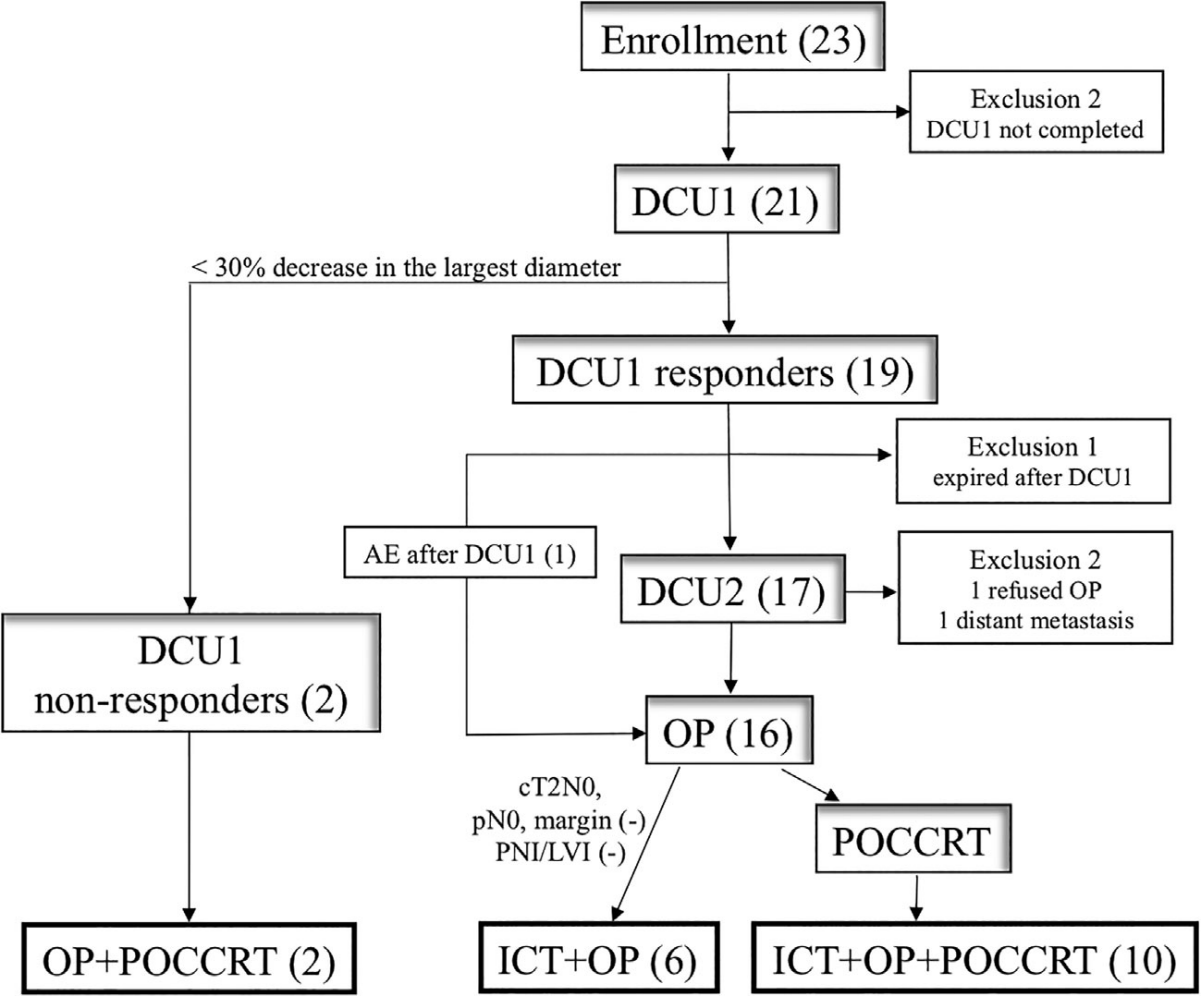
Flow diagram of patient enrollment and distribution into treatment groups. Abbreviations: OP, operation, ICT, induction chemotherapy, POCCRT, postoperative adjuvant chemoradiotherapy, AE, adverse events, PNI, perineural invasion, LVI, lymphovascular invasion, TE, tumor emboli. The number in the parentheses represents the number of patients

A total of 23 patients were enrolled from July 2011 to December 2015 (Table 1); among whom 13 patients (56.5%) had cT3–4 tumors, 18 (78.2%) had clinical N+ disease, and 20 (87%) were classified as stage III–IV. Patient enrollment and distribution are summarized in Figure 2. Two patients dropped out without completing DCU1 at their decision because of nontolerable nausea and Port‐A catheter infection, respectively. Nineteen (90.5%) of the 21 patients evaluable for DCU1 were determined as DCU1 responders (Table 2).

**TABLE 1:** Characteristics of all enrolled patients (*n* = 23)

	No. of patients (%)
Age, years, mean (range)	52.3 (30–69)
Gender	
Male	22 (95.7)
Female	1 (4.3)
Cigarette smoking	
Current	15 (65.2)
Former	5 (21.7)
Nonsmoker	3 (13.0)
T classification^a^	
cT2	10 (43.5)
cT3	2 (8.7)
cT4	11 (47.8)
N classification^a^	
cN0	5 (21.7)
cN1	3 (13.0)
cN2b	7 (30.4)
cN2c	8 (34.8)
Overall stage^a^	
II	3 (13.0)
III	2 (8.7)
IV	18 (78.3)

^a^ According to the 7th edition of the American Joint Committee on Cancer.

**TABLE 2:** Induction chemotherapy response evaluation

	No. (%)
DCU1 (*n* = 21)	
Responders	19 (90.5)
Nonresponders	2 (9.5)
DCU2 (*n* = 17)	
Responders	
Partial response	8 (47.1)
Complete response	7 (41.2)
Nonresponders	
Progressive disease	1 (5.9)
Stable disease	1 (5.9)

*Note*: DCU1 the first cycle of induction chemotherapy. DCU2 the second cycle of induction chemotherapy.

After DCU1, one patient developed severe adverse events (SAE) with liver function deterioration and ascites despite a good response, and immediate surgery was arranged. Another patient died from febrile neutropenia and sepsis. Seventeen patients completed the entire course of DCU2. One patient refused scheduled surgery after DCU2, and another developed lung metastasis after surgery; these two patients were excluded from subsequent analyzes. Among the 17 included patients, 15 (88.2%) were determined as clinical DCU2 responders (Table 2).

Ultimately, 16 patients underwent surgery, one after DCU1 and 15 after DCU2; POCCRT was waived in 6 patients according to our risk‐adapted criteria.

### 3.2 Safety and adverse events of DCU induction chemotherapy

Cumulative severe hematologic and nonhematologic toxicities or SAE (grade 3–4) during DCU1 and/or DCU 2 are listed in Table 3. Grade 3 to 4 leukopenia being the most common SAE (42.9%, 9/21), and grade 4 neutropenia (< 500/mm^3^) occurred in two patients. One patient had early signs of sepsis after DCU1, which required intensive care; the patient then underwent DCU2 smoothly, with an 80% adjustment in dose reduction. Another patient with multiple comorbidities, including stroke, myocardial infarction, and diabetes mellitus, expired after DCU1 due to intractable febrile neutropenia and sepsis. Nonhematologic SAEs occurred less frequently. Nausea and oral mucositis (19% and 14.3%, respectively) were the two most commonly observed nonhematologic SAEs.

**TABLE 3:** Adverse events during induction chemotherapy (*n* = 21)

AE category	Grade	No. of cases (%)
Hematologic		
Leukopenia	All grades	14 (66.7)
	Grade ≥ 3	9 (42.9)
Febrile neutropenia^a^	All grades	2 (9.5)
	Grade ≥ 3	2 (9.5)
Anemia	All grades	15 (71.4)
	Grade ≥ 3	1 (4.8)
Thrombocytopenia	All grades	5 (23.8)
	Grade ≥ 3	2 (9.5)
Nonhematologic		
Anorexia	All grades	6 (28.6)
	Grade ≥ 3	2 (9.5)
Nausea	All grades	14 (66.7)
	Grade ≥ 3	4 (19.0)
Vomiting	All grades	10 (47.6)
	Grade ≥ 3	2 (9.5)
Oral mucositis	All grades	9 (42.9)
	Grade ≥ 3	3 (14.3)
ALT increased	All grades	4 (19.0)
	Grade ≥ 3	0 (0)
Acute kidney injury	All grades	3 (14.3)
	Grade ≥ 3	0 (0)
Diarrhea	All grades	9 (42.9)
	Grade ≥ 3	2 (9.5)
Constipation	All grades	3 (14.3)
	Grade ≥ 3	0 (0)
Skin rash	All grades	1 (4.8)
	Grade ≥ 3	0 (0)
Alopecia^a^	All grades	4 (19.0)
	Grade ≥ 3	–

*Note*: AE, adverse events; ALT, alanine aminotransferase.

^a^ According to the Common Terminology Criteria for Adverse Events version 4.0, there were no definitions of grade 1/2 febrile neutropenia and grade 3/4 alopecia.

### 3.3 Surgery for residual tongue tumor and adjuvant therapy

Surgery was performed in 16 responders to ICT, 1 after DCU1 and 15 after DCU2. Ten patients (62.5%) received transoral excision without the need for flap reconstruction after ICT; this approach was only feasible in five patients (31.25%) after initial evaluation by the responsible surgeons. Of the five patients who avoided the mandibulotomy approach and flap reconstruction, four had cT4a tumors at diagnosis.

The pathologic results are summarized in Table 4. Pathologic complete response (pCR) of primary tongue cancer was determined in eight patients (50%), one after DCU1 and seven after DCU2. In 7 of the 17 patients (41.2%) who completed DCU2, pCR was achieved despite being initially determined as partial response (PR) on MRI. It is noteworthy that one DCU1 responder who received immediate surgery without DCU2 (owing to AE) also achieved pCR. With regards to the eight patients with pathologic PR, only one (12.5%) had a positive surgical margin. The margin positive rate of all 16 patients who received conservation surgery was 6.3% (1/16). Pathologic lymph node metastasis (ypN+) was documented in four patients (25%) after ICT, although cN+ was determined in 12 patients (75%) at initial diagnosis.

**TABLE 4:** Pathologic results in 16 responders to induction chemotherapy

stage^a^	N0	N1	N2c	Total (%)
ypT0	8^b^	0	0	8 (50.0)
ypT1	1	0	0	1 (6.3)
ypT2	3	2	1	6 (37.5)
ypT3	0	0	1	1 (6.3)
Total (%)	12 (75.0)	2 (12.5)	2 (12.5)	16 (100)

^a^ According to the 7th edition of the American Joint Committee on Cancer.

^b^ Including 1 patient underwent operation after adverse events of DCU1.

POCCRT was not applied by the risk‐adapted criteria in 6 patients, including the patients who received only DCU1 and achieved pathologic CR. The remaining 10 patients (62.5%) received POCCRT with a mean CTV radiation dose of 6000 ± 589 cGy (range, 5000–6600 cGy) concomitantly with a mean of six cycles of weekly chemotherapy with cisplatin administration (range, 5–7 cycles).

### 3.4 Oncological and functional outcomes

**FIGURE 3:**
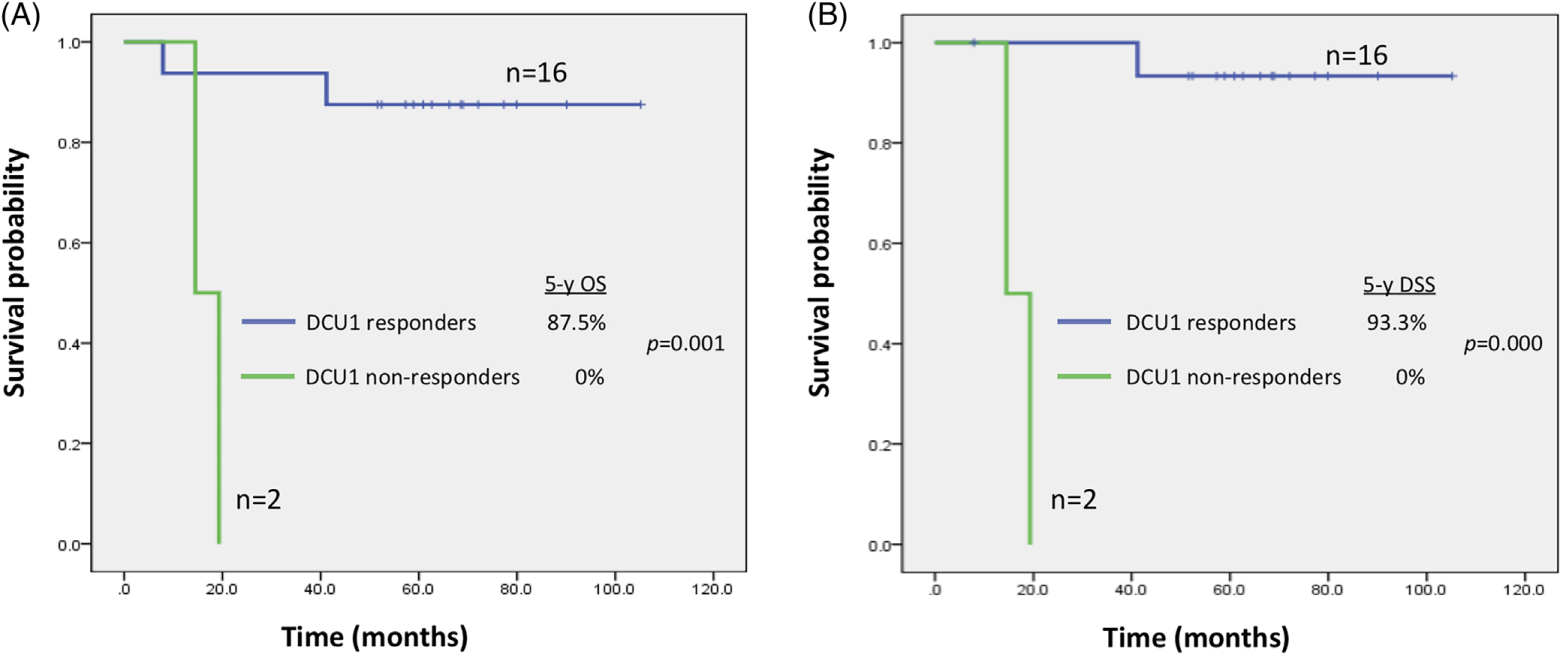
Kaplan–Meier curves for (A) overall survival and (B) disease‐specific survival for patients stratified by tumor response to the first course of induction chemotherapy. Abbreviations: OS, overall survival; DSS, disease‐specific survival

Of the 23 intent‐to‐treat subjects in our study, the 5‐year OS and DSS rates were 67.0% and 70.7%, with a median follow‐up duration of 57.3 months (range, 0.5–105.2 months). Excluding the five patients who dropped out, 18 patients were clinically followed and categorized into three groups according to treatment course: the DCU1 nonresponders, ICT + operation (OP), and ICT + OP + POCCRT (Figure 2). The median follow‐up duration was 58.6 months (range, 7.9–105.2 months), and the 5‐year OS and DSS rates were 77.8% and 82.4%, respectively. When the DCU1 nonresponder group was compared to the latter two groups (DCU1 responders), a significant difference in 5‐year OS (0% vs. 87.5%, *P* = 0.001) and DSS (0% vs. 93.3%, *P =* 0.000) were observed (Figure 3).

For the DCU1 nonresponders, patients were initially staged as cT4aN0M0 and cT4aN2bM0. Pathologic analysis revealed ypT3N0 and ypT3N2b in two patients, with one positive margin. Both patients received POCCRT with an accumulative dose of 6600 cGy. However, early local recurrence developed at 3 and 9 months after treatment, and they expired 11 and 14 months after POCCRT, respectively. Similarly to the poor prognosis of DCU1 nonresponders, the only patient who had a poor response to DCU2 with the stable disease showed a dismal prognosis despite no clear evidence of disease.

For the DCU1 responders, the ICT + OP group consisted of six patients and the ICT + OP + POCCRT group comprised 10 cases. In the ICT + OP group, one patient received only DCU1, and all six patients were disease‐free, with a mean follow‐up time of 63.9 months (range, 52.4‐79.9 months). Of the 10 patients in the ICT + OP + POCCRT group, eight remained disease‐free after a mean follow‐up of 63.8 months (range, 7.9‐105.2 months), and two disease‐related deaths occurred during the follow‐up period. One of the patients was a DCU2 nonresponder and died from aspiration pneumonia complicated with septic shock 3 months after POCCRT, with no evidence of recurrence. The other patient died from a local recurrence that occurred 27 months after POCCRT. No distant metastasis was observed in the latter two groups, and functional outcomes were satisfactory. Neither feeding tube nor tracheostomy was required after treatment in any of the 14 disease‐free patients.

## 4 DISCUSSION

There has been a paucity of data about chemoradiotherapy in OSCC due to the poorer response and inferior outcome compared with surgery.^5^, ^6^ Our results of this phase II trial suggest that conservation surgery after ICT is feasible in resectable OTSCC and may lower the need for mandibulotomy, free flap reconstruction, and adjuvant chemoradiotherapy. Good oncological outcomes can be observed in ICT responders. The DCU regimen in this study was easy to administer and carried a low rate of SAE.

Several studies have reported the use of ICT with different regimens in OSCC patients. Zhong et al.^17^ reported a phase III trial of ICT with TPF followed by surgery in locally advanced OSCC, in which they achieved a clinical response rate of 80.6% after two cycles of TPF, including a pCR rate of 13.4%. Licitra et al.^16^ also reported a clinical response rate of 82% and a pCR rate of 27% with a PF regimen (cisplatin and fluorouracil) in OSCC. Compared with these two studies, the DCU regimen in our study achieved better response rates (90.5% and 88.3% for DCU1 and DCU2, respectively) (Table 2) and an impressive pCR rate (50%). These positive results may partly be explained by the fact that the DCU regimen was used in a patient group with a substantial proportion of cT2 tumors (43.5%).

In our study, daily oral UFUR was given at an equivalent dose to ameliorate stomatitis induced by the continuous 5‐fluorouracial infusions of the TPF regimen.^1^, ^26^, ^27^ The lower rate of grade 3–4 mucositis (14.3%) and convenient administration without the need for hospitalization also improved patient compliance. The mean clearance of docetaxel has been reported to be lower in Asian populations than in Caucasian populations.^28^, ^29^ Therefore, a dose modification with docetaxel in Asian populations had been used in the treatment of various kinds of cancers.^30^, ^31^, ^32^, ^33^ In our study, grade 3/4 leukopenia and febrile neutropenia were noted in 42.9% and 9.5% of the patients, respectively, slightly higher than those reported in the studies using ICT with TPF^13^, ^33^, ^34^; a larger accumulative dose of docetaxel in one cycle of ICT compared to other Asian studies (72 mg/m^2^ vs 60 mg/m^2^) may be the cause of this difference. Despite the myelotoxicities, more than 70% (17 out of 23 patients) completed two cycles of ICT, suggesting good compliance to the regimen. In our experience, toxicities of the DCU regimen occurred gradually with dose accumulation and were less aggressive or fulminant compared to TPF. However, an old‐aged patient with multiple comorbidities expired after one cycle of ICT owing to intractable febrile neutropenia and subsequent sepsis. Therefore, ICT with the DCU regimen should still be administrated carefully, especially in patients with high comorbidities.

With regard to oncological outcomes, we found a significant difference between DCU1 nonresponders and responders in both 5‐year OS (0% vs. 87.5%, *P* = 0.001) and DSS (0% vs. 93.3%, *P* = 0.000). Of the 16 responders who underwent subsequent surgery with or without adjuvant therapy, only one developed local recurrence (6%, 1/16). In contrast, both DCU1 nonresponders had dismal survival outcomes from rapid local recurrence, despite the fact that surgery with adjuvant therapy was arranged immediately. Inhestern et al.^35^ showed significantly better progression‐free survival and OS rates in responders to TPF induction before surgery in advanced oral and oropharyngeal cancers. Zhong et al.^17^ also reported a similar result of better OS and locoregional control in patients with advanced OSCC who had a favorable clinical or pathologic response to ICT. Our results further suggest a chemoselection effect for patients with favorable oncologic outcomes with just one cycle of ICT; however, management of DCU nonresponders remains problematic in this study.

The principle of surgery after chemotherapy or radiotherapy in HNSCC often advocates excision by the original tumor extent due to concerns regarding nonconcentric tumor shrinkage.^36^, ^37^ However, conservation surgery has been safely applied after neoadjuvant chemotherapy in breast cancer patients to optimize oncological and cosmetic outcomes.^38^, ^39^, ^40^, ^41^ In this study, similar to breast conservation surgery, the extent of surgical excision was advocated according to the size of residual lesion after ICT. Transoral surgery, without the need for mandibulotomy or flap reconstruction, was performed in 62.5% of patients, in contrast to 31.5% at initial surgical planning at diagnosis (Table 4). Notably, of the five patients who waived the mandibulotomy approach and flap reconstruction, four had cT4a tumors. The margin positive rate under this surgical principle was 6.3%. Only one patient developed local recurrence 27 months after POCCRT, implying that surgery according to residual tumor after ICT may be feasible and safe in selected OTSCC patients. We excluded tumors with gingiva or mandible involvement due to the possibility of tumor adherence on the bone or periosteum, leading to nonconcentric tumor shrinkage after ICT. Further studies should be carried out to verify this hypothesis.

The limitation of our study is the relative small sample size. Since this was a phase II trial, more patients should be recruited with a comparison group in the future study. In addition, DCU1 nonresponders had extremely poor oncologic outcomes. Treatment delay from ineffective ICT in nonresponders, although determined after just one cycle of DCU, is also a concern. Therefore, molecular markers should be further investigated to predict the response to ICT and allow proper selection of candidates for ICT. Until then, the benefit of ICT may be fully exploited in responders, and nonresponders will be spared from treatment delay and toxicities. Finally, functional outcomes, other than tracheostomy and tube feeding, were not addressed in this study. The details of functional improvement and quality of life require further study.

## 5 CONCLUSION

In conclusion, tongue conservation treatment by ICT with a DCU regimen, followed by surgery and risk‐adapted adjuvant therapy, is feasible for OTSCC. A good oncological outcome could be achieved in ICT responders with risk‐adapted adjuvant therapy. However, the outcomes of nonresponders are dismal. Further study in a larger patient population with a control group is required to explore the oncologic and functional benefit of this treatment.

## CONFLICT OF INTEREST

The authors declare that they have no potential conflicts of interest.

## AUTHOR CONTRIBUTIONS

**Tsung‐Lun Lee:** Data curation; resources; writing‐original draft. **Pei‐Yin Wei:** Writing‐original draft. **Muh‐Hwa Yang:** Methodology; resources; supervision. **Peter Mu‐Hsin Chang:** Resources. **Ling‐Wei Wang:** Resources. **Shyh‐Kuan Tai:** Conceptualization; funding acquisition; methodology; project administration; supervision; writing‐review & editing.

## ETHICS STATEMENT

All procedures performed in studies involving human participant were in accordance with the ethical standards of the institutional research committee and with the 1964 Helsinki declaration and its later amendments or comparable ethical standards. Signed informed consent was obtained from patients.

## Data Availability

I confirm that I have included a citation for available data in my references section.
